# Prostate Biopsy Sampling Causes Hematogenous Dissemination of Epithelial Cellular Material

**DOI:** 10.1155/2014/707529

**Published:** 2014-01-28

**Authors:** Sam Ladjevardi, Gert Auer, Juan Castro, Christer Ericsson, Anders Zetterberg, Michael Häggman, Hans Wiksell, Håkan Jorulf

**Affiliations:** ^1^Surgical Science, Uppsala University, 751 85 Uppsala, Sweden; ^2^Karolinska Biomic Center, Karolinska Institutet, 171 76 Stockholm, Sweden; ^3^Department of Oncology-Pathology, CCK R8:04, Karolinska Institutet, 171 76 Stockholm, Sweden; ^4^Department of Molecular Medicine and Surgery, Karolinska Institutet, c/o Hans Wiksell, Comair AB, P.O. Box 39011, 100 54 Stockholm, Sweden; ^5^Radiology, Oncology and Radiation Science, Section of Radiology, Uppsala University, 751 85 Uppsala, Sweden

## Abstract

The extent of epithelial cellular material (ECM) occurring in venous blood samples after diagnostic core needle biopsy (CNB) was studied in 23 patients with CNB diagnosed prostate cancer without provable metastases and 15 patients without cancer. The data show a significant increase of ECM in the peripheral blood sampled 20 seconds or 30 minutes after the last of 10 CNB procedures compared to the number of ECM detectable in the blood samples taken before the performance of CNB. The data indicate that diagnostic CNB of prostate cancer causes an extensive tissue trauma with a potential risk of cancer cell dissemination.

## 1. Introduction 

Prostate cancer (PCa) is one of the most common malignant tumors in western countries and the second common cause of death in men [1]. The American Cancer Society estimates that in 2013, 238,590 new cases of prostate cancer will be diagnosed in the United States and 29,720 people will die of the disease [2]. With prostate-specific antigen (PSA) screening, most potential prostate cancers are diagnosed at an early stage [3, 4]. The 10-year relative survival with well-differentiated PCa has been shown to be 100% regardless of treatment [5]. The 5-year relative survival rate for all stages of prostate cancer is 98%, which indicates that prostate tumours develop slowly and survival is hardly affected [6, 7]. However, about a third is aggressive and may metastasize. The choice of treatment depends on the patient's age at diagnosis, the stage and perceived aggressiveness of the tumour, the potential side-effects of treatment, and patient comorbidity [8–10].

PCa is usually multifocal but in the currently common case-mix, only a fraction of the prostate is typically affected by cancer, which has made multiple transrectal ultrasound (TRUS) biopsies essential to assure well-representative samples. A series of core needle biopsies (CNB, 18 G, and 1.25 mm diameter) are taken according to a routine scheme, 5–8 biopsies from each side under ultrasound guidance. Histologic diagnosis is performed according to the Gleason score defining the two most common cell growth patterns in the specimen. However, typically 10 routine biopsies can sample approximately only 0.2% of the prostate volume and therefore may not be representative of the entire cancer morphology panorama, even though the cancer detection rate may be 30–40%. Targeted biopsy of the most suspicious areas is possible after magnetic resonance imaging (MRI), diffusion weighted (DWI), and 2D MR spectroscopic imaging (MRSI) by demonstrating differences in apparent diffusion coefficients, ADC [11].

Metastases locate particularly to the local lymph nodes and to bones. The local lymph node spread is due to extra cellular fluid draining through the lymph nodes. The spread beyond the lymph nodes, to bones and other secondary organs, is hematogenic. The location of distant metastases may be determined by a combination of the circulatory patterns, the properties of the seeding cells, and the microenvironment in the secondary organ.

Epithelial cellular material dissemination is seen in the peripheral blood samples of prostate cancer patients [12]. These may correspond to cells or microparticles that have extravasated from the prostate, even among these patients selected for having no detectable distant metastases. They may be a trauma indicator as well as a potential risk factor for disseminating the disease.

At CNB of the prostate, there is an obvious risk of dissemination of cell material to surrounding tissue and blood and lymph vessels. Thus there exists a need for improved, imaging-guided, biopsy procedures to limit the trauma and to focus the biopsies to the most aggressive area of possible PCa, to limit the number of biopsies, and to take whatever steps that are possible to inhibit or eliminate dissemination of ECM.

### 1.1. Aim of the Study

Multiple biopsies of the prostate for routine diagnosis may cause the release of cancer cells or subcellular material. There exists a possibility that the released material may spread the cancer not only locally but also might cause distant metastases.

The aim of this study was to analyze peripheral venous blood for possible cellular biomarkers from prostate tissue in order to analyze any possible spread of epithelial cells, cancer cells or subcellular material, released during the CNB procedure.

## 2. Material and Methods

### 2.1. Patient Inclusion

Between 2010 and 2011 we included 45 men admitted to the urology department for high level of prostate specific antigen (PSA). Men up to 75 years old and 3.0 < PSA ≤ 20 ng/mL, or suspected tumour on digital rectal examination, were included. The study was approved by the Ethical Committee of Uppsala Sweden number 2009/191. Informed consent was obtained. Patient data are presented in Tables 1 and 2.

All men initially underwent standard 18 G (1.25 mm) ultrasound guided spring loded CNB according to local hospital routine, five on each side: one apex, one medial and lateral middle zone, and one medial and lateral from the base.

Of these 45 men, 24 turned out to present with PCa, Gleason score min. 3 + 3 at CNB. One patient did not complete the study. The 23 patients positive for PCa at CNB were included in the study (Table 1, group A), as were 15 of 21 patients who were negative for PCa at CNB (Table 1, group B). Six patients negative for PCa were not included due to not consenting or due to technical failure in sampling peripheral venous blood after CNB.

Ten of the patients positive for PC underwent laparoscopic radical prostatectomy (LRP, Table 2, group C); 2 underwent radiotherapy (RT, group D). Eleven patients received active surveillance (group E) based on a small cancer volume and highly differentiated cancer at CNB. Metastatic disease to pelvic lymph node was demonstrated in one patient who had radiotherapy.

There was only a slight difference in age between groups A and B, 65 and 62 years, respectively. The mean PSA was 8 compared to 5.3 ng/mL. Patients with PCa group A had larger CNB volume than the control group, mean 90.8 mm³ compared to 71.2 mm³.

There was no significant difference in Gleason scores between the groups C, D, and E: mean 7, 8.5, and 6, respectively (Table 2).

### 2.2. Blood Sampling

Of the 45 initially included patients, blood sampling procedure was successful before and after CNB of the prostate in altogether 38 patients that is, 23 patients, with PCa (Table 1, group A) and 15 patients (control group) with no PCa diagnosed by routine CNB (Table 1, group B).

Sampling of 10 mL peripheral venous blood (Vacutainer EDTA) from the arm vein was obtained immediately before CNB. In 8 of 23 patients in group A, blood sampling after CNB was performed within 20 seconds after the last CNB and in 15 of the 23 patients after 30 minutes. The corresponding numbers in group B were 4 patients after 20 seconds and 11 patients 30 minutes after the last CNB. The 10 CNB were taken within 2 minutes and 40 seconds to 4 minutes and 50 seconds.

Peripheral venous blood samples were kept in room temperature during tests and transport. Processing of the blood samples was performed by centrifugation at 2000 rpm/5 min. Plasma was decanted and replaced by 10 mL PBS and 1 mL 4% formalin and were transported, with constant rocking, to the Cytology Laboratory at the Cancer Center Karolinska (CCK) at the Karolinska Institute for analysis.

### 2.3. Epithelial Cellular Material Analysis

Peripheral blood samples were taken in EDTA tubes. The first sample was not used for blood analysis in order to reduce the amount of epithelial cellular material from the skin when the blood sampling needle was placed in the arm vein. The blood was processed through a prototype liquid biopsy cell sample preparation instrument as previously described [13]. The resulting concentrated cellular material was suspended in proprietary buffer, sedimented onto slides, washed, and stained using the anti-cytokeratin antibody anti-CD45 and DAPI. EPM was visualized using either a bright field or a confocal microscope.

### 2.4. Calculation of CNB Volume

Core needle 18 G (outer diameter 1.25 mm) was used with total slot volume 4.72 mm³ (total length of the slot = 19.2 mm; *r* = 0.475 mm; height of solid base in the slot = 0.36 mm). The pathologists reported the length of each biopsy sample in mm and also the length of any cancer fraction in the respective sample. We based our calculations on that the biopsy sample was filled for each sample length. From these values, the total biopsy volumes and cancer volumes were calculated for each of the 10 CNB sampling procedures.

### 2.5. Evaluation Criteria for Circulating Epithelial Cellular Material

Cytokeratin positive, CD45 negative cellular material, was considered indicative of hematogenous epithelial cellular material. Those of 10–20 *μ*m size and with DAPI positive cell nuclei may be classified as representing circulating tumor cells (CTCs), while those with a smaller size and lacking a DAPI detectable cell nucleus were classified as epithelial cellular material.

## 3. Results

### 3.1. Biomarkers for PCa

PSA levels were higher in the CNB PCa group (Table 1; mean 8 ng/mL) compared to the non-PCa group (5.3 ng/mL) (Table 1). The Gleason score was only slightly higher in patients who had treatment, prostatectomy (*N* = 10), or radiation (*N* = 2), mean 7.3 compared to 6 for those who had active monitoring (*N* = 11).

### 3.2. CNB Volume/Share of Prostate Cancer Volume

Calculated from postoperative serial sections, cancer volume in 10 patients who had prostatectomy was 4.3 cm³ (1.2–8.4 cm³) representing 3.6–23.5% of the prostate volume (Table 2). However, the total volume from the 10 CNB in these 10 patients was small, 1.3–34 mm³, representing on average only approximately 0.2% of the prostate volume. The fraction of the cancer tissue in these 10 CNB was on average 13.6%, with a range 0.3–84%. The CNB cancer volumes were generally small in all groups C, D, and E (Table 2) representing from only 1/1000 (group D) and 1/2800 (group C) to 1/21000 (group E) share of the calculated prostate volumes. Thus a very small but variable share of a CNB may represent cancer tissue on routine 10 CNB.

### 3.3. Epithelial Cellular Material

**Figure 1:**
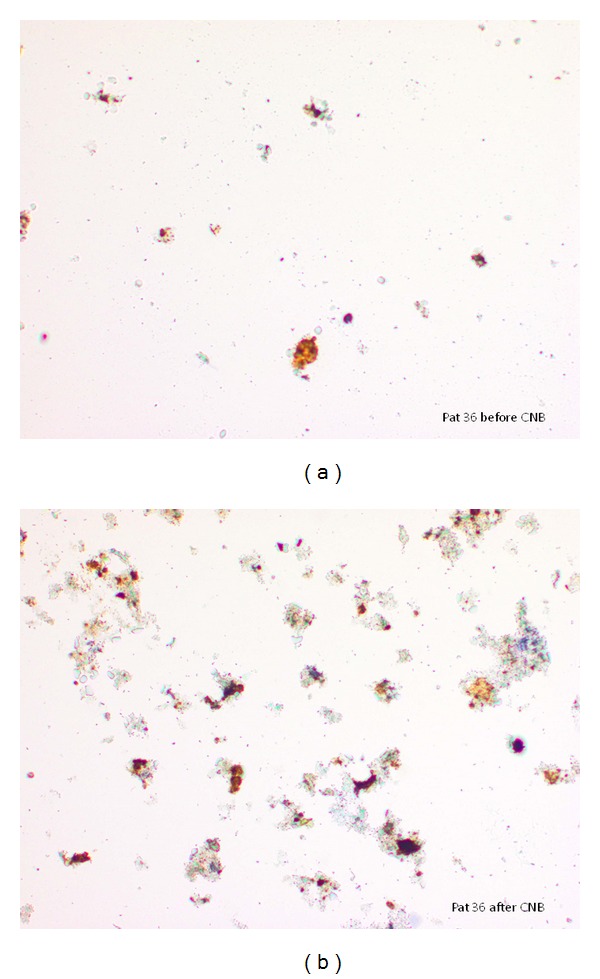
(a) Age 70. PSA 13.0 ng/ml; Gleason score 3 + 4; prostate volume 26.5 cm³, cancer volume 4.3 cm³ at serial sections. Peripheral venous blood sample before CNB. Epithelial cellular material categorized score 2. (b) Same patient as (a). Peripheral venous blood sample 30 min after CNB. Epithelial cellular material score 3.

Epithelial cellular material dissemination in the peripheral blood was demonstrated before CNB in 9/23 patients diagnosed for PCa at routine CNB, and in 1/15 patients who were negative for PCa at CNB (Table 1). Epithelial cellular material was demonstrated in peripheral venous arm blood after CNB in 19/23 patients in group A (Figure 1) and in 2/15 patients in group B (Tables 1 and 3) (Figure 1). Blood sampling was performed using the same indwelling cannula before and after tissue sampling.

In the corresponding numbers in group B, sampling within 20 sec after the last CNB, none of 4 patients had increased epithelial cellular material in peripheral venous arm blood. When blood sampling was performed 30 min after the last CNB, 1 of 11 had epithelial cellular material in peripheral venous blood before CNB and 2 after CNB in this group (Table 1).

There is a significant increase of epithelial cellular material after CNB in patients of group A, whether blood sampling was performed within 20 sec after the last CNB (5 of 8), or after 30 min (9 of 15). There was no significant difference in increase of score between group A, who received radical prostatectomy (6 of 10), or active surveillance (6 of 11). Both patients who received radiotherapy had increased score after CNB (Tables 2 and 3). None of the patients in the whole cohort had decreased epithelial cellular material after CNB.

## 4. Statistics

1. PCa at CNB (*N* = 23): patients had prostatectomy (*N* = 10), radiation (*N* = 2), or active surveillance (*N* = 11).
  - 9/23 patients no changes in epithelial cellular material score after CNB (excluded from calculation).
  - 14/23 patients had increased epithelial cellular material score after CNB.
2. No PCa at CNB (*N* = 15). No treatment or active surveillance.
  - 14/15 patients no changes in epithelial cellular material score after CNB (excluded from calculation).
  - 1/15 patient had increased epithelial cellular material score after CNB.
3. Difference between (1) and (2): Mann-Whitney *U*-test significantly increased epithelial cellular material score in group A compared to group B, Mann-Whitney *U*-test, *P* < 0.002.

## 5. Discussion

There is a clear correlation between the presence of epithelial cellular material in peripheral venous blood and the presence of prostate cancer. Furthermore, epithelial cellular material increases in peripheral venous blood after routine CNB (10 biopsies 18 G) of the prostate in men with prostate cancer diagnosed with elevated Gleason score. In this material, epithelial cellular material increased from 39% in the included 23 patients with PCa Gleason mean 6.4 (5–10) to 82% after routine series 10 CNB. In 15 patients negative for PCa after routine 10 CNB but PSA mean 5.3, one patient had epithelial cellular material before and two patients after CNB. In this small material, there was no difference in epithelial cellular material if peripheral venous blood sampling was performed 20 seconds or 30 minutes after the last CNB. Thus we do not have strong evidence for a rapid clearing of epithelial cellular material in the time interval 20 seconds to 30 minutes. The present epithelial cellular material was defined by their cytokeratin positivity, which in turn means that the cells had not undergone significant epithelial to mesenchymal transition (EMT) and are expected to be relatively stiff. Nevertheless some material, especially the small sized cellular material, obviously can pass the capillary network and reach to the arm vein.

It is interesting that some patients show circulating epithelial cellular material before CNB demonstrating that other causes, such as palpation, may contribute to epithelial cellular material dissemination.

It is also interesting that some patients without PCa had epithelial cellular material, demonstrating that even nonmalignant or premalignant conditions may cause dissemination of epithelial cellular material. It is a limitation of this study that there is no molecular studies of the epithelial cellular material, except for the criteria used to identify them. Further molecular studies of the epithelial cellular material in the different conditions may reveal means to distinguish the non- or pre-malignant process from the malignant process.

The spring loaded 18 G needle penetrates the prostate tissue with an average speed of approximately 3–8 m/s thus causing significant risk for local trauma releasing epithelial cells or cell material into surrounding tissue, lymph, or blood vessels.

Typically CNB will represent a very small part of the prostate, approximately 0.2%, while the total cancer volume retrieved from the needle usually is only 1–5 mm³. However, one needle pass 20–25 mm long is traumatizing the tissue with high speed and the potential to cut and release cell material.

The number of cancer positive needle passes is typically 1 to 5. In our material, the mean number of cancer positive needle passes was 2.5 (1–10). Thus the majority of biopsy needles will not contain cancer material, indicating the need for better hit rate. This is now possible by MRI imaging of the prostate identifying the area of highest cancer rate for targeted biopsy [11, 14], which in turn can contribute to reduce the number of CNB and thus the risk of cancer cell dissemination.

## Figures and Tables

**Table 1:** Patient data, positive (A) or negative (B) for PCa at routine CNB. Number of patients with epithelial cellular material in peripheral venous blood demonstrated before and after CNB; and increased score after CNB.

Mean and range within brackets	Group A CNB pos. for PCa patients (*N* = 23) mean, range	Group B CNB neg. for PCa patients (*N* = 15) mean, range P
Patient age	65 (50–75)	62 (35–72)
PSA ng/mL	8 (3.8–17)	5.3 (0.9–epithelial cellular material)
CNB volume mm^3^	90.8 (71.9–117.2)	71.2 (56.3–96.3)
CNB Ca/CNB Vol%	13.6 (0.3–84)	Not available
Gleason score	6.4 (5–10)	Not available
Prostate vol cm^3^ Way of calculation	40.5 (16–75) Postop serial sections	52.7 (29–101) TRUS
Epithelial cellular material		
Before CBN	9 patients	1 patient
After CNB	19 patients	2 patients
Increased score	14 patients	1 patient

**Table 2:** Patients group A (*N* = 23) with PCa, way of treatment. CNB and prostate data. Number of patients with epithelial cellular material in peripheral blood before and after CNB and increased score. Individuals in each group C, D, E are also presented in Table 3.

Group A = C + D + E vol = volume P = prostate	C PCa prostatectomy (*N* = 10)	D PCa radiation (*N* = 2)	E PCa active surveillance (*N* = 11)
CNB vol mm^3^	85.1 (71.9–107.2)	100.6 (95.1–106)	97 (72–117.2)
CNB vol/P vol	1/510	1/480	1/620
CNB PCa vol mm^3^ mean and range share of P vol	13.2 (1.3–34) mean 1/2800	46.4 (4.3–89) mean 1/1000	2.7 (0.3–13.9) mean 1/21000
CNB Ca vol/CNB vol, %	19 (1.1–25.3)	40 (5–84)	3 (0.3–14)
Gleason score	7 (6–8)	8.5 (7–10)	6 (5–7)
P vol cm^3^ Way of calculation	32.2 (24–46.9) Postop.	41.5 (40–43) TRUS	47.9 (16–75) TRUS
PCa vol from postoperative serial sections, cm^3^	4.3 (1.2–8.4) (3.6–23.5%, mean 13.4% of P vol.)	Not available	Not available
Epithelial cellular material			
Before CBN	4 patients	0 patients	5 patients
After CNB	9 patients	2 patients	8 patients
Increased score	6 patients	2 patients	6 patients

**Table 3:** Patients with PCa at CNB, way of treatment (groups C, D, and E). Score for epithelial cellular material in peripheral venous blood before/after CNB: 0 = none; 1 = 1–5; 2 = 6–10; 3 = 11, and more. Change of score before/after CNB.

Pat number	PCa at CNB	Op = 1 not = 0	Epithelial cellular material before CNB score	Epithelial cellular material after CNB score	Epithelial cellular material difference before/after CNB score change
Prostatectomy (C)					
1	1	1	0	1	1
2	1	1	0	1	1
3	1	1	0	0	0
4	1	1	0	1	1
5	1	1	2	2	0
6	1	1	2	2	0
7	1	1	1	1	0
8	1	1	0	1	1
9	1	1	2	3	1
10	1	1	0	1	1
*∑*	10	10	4	9	6

Radiation treatment (D)					
14	1	0	0	1	1
20	1	0	0	1	1
*∑*	2	2	0	2	2

Active surveillance (E)					
11	1	0	0	1	1
12	1	0	0	0	0
13	1	0	0	0	0
15	1	0	0	0	0
16	1	0	0	1	1
17	1	0	0	2	1
18	1	0	2	2	0
19	1	0	2	3	1
21	1	0	1	2	1
22	1	0	1	2	1
23	1	0	3	3	0
*∑*	11	11	5	8	6

All patients with PCA at CNB					
*∑*23	23		9	19	14
